# Effects of acute stress on probabilistic reversal learning in healthy participants

**DOI:** 10.1192/j.eurpsy.2021.1956

**Published:** 2021-08-13

**Authors:** L. Wieland, C. Ebrahimi, T. Katthagen, M. Panitz, A. Heinz, Z. Sjoerds, F. Schlagenhauf

**Affiliations:** 1 Psychiatry And Psychotherapy, Charité Universitätsmedizin, Berlin, Germany; 2 Neurologie, Max-Planck-Institut für Kognitions- und Neurowissenschaften, Leipzig, Germany; 3 Institute Of Psychology - Cognitive Psychology Unit, Leiden University, Leiden, Netherlands

**Keywords:** stress, reward, learning

## Abstract

**Introduction:**

Altered reward-based learning and stress play an important role in psychiatric illnesses, such as psychosis or addiction. Stress sometimes increases learning from rewards, other times it does not show an effect (Starcke & Brand, 2016). A task addressing reward-based learning is the reversal learning task, which uses probabilistic rewards as feedback and incorporates sudden changes in reward contingencies. The effects of acute stress on reversal learning have rarely been addressed.

**Objectives:**

Here, we investigated the effect of acute social stress in a within-subject design in healthy participants.

**Methods:**

A sample of n = 28 male non-clinical participants performed the task in a control condition versus the Trier Social Stress Test (TSST), a validated method to induce psychosocial stress. In our version of the reversal learning task (Reiter, 2016), participants choose between two anti-correlated stimuli in order to obtain rewards in three blocks. Reward contingencies remain stable for the first 55 trials and the last 35 trials. During the second block, in between the stable blocks, four changes of reward contingencies require participants to flexibly adapt their behavior. Performance was measured in correct responses, switches after losses and wins.

**Results:**

Cortisol and subjective stress responses showed that the stress induction was successful. Preliminary analyses showed no significant effect of stress induction on any of the performance measures.

**Conclusions:**

These results demonstrate that reversal learning, at least regarding overall performance measures in our task, is robust to stress-related changes. Modeling and fMRI analyses could yield further insights into more subtle changes after stress induction.

**Disclosure:**

No significant relationships.

